# A Permutation Method to Assess Heterogeneity in External Validation for Risk Prediction Models

**DOI:** 10.1371/journal.pone.0116957

**Published:** 2015-01-21

**Authors:** Ling-Yi Wang, Wen-Chung Lee

**Affiliations:** 1 Research Center for Genes, Environment and Human Health and Institute of Epidemiology and Preventive Medicine, College of Public Health, National Taiwan University, Taipei, Taiwan; 2 Department of Medical Research, Tzu Chi General Hospital, Hualien, Taiwan; University of Westminster, UNITED KINGDOM

## Abstract

The value of a developed prediction model depends on its performance outside the development sample. The key is therefore to externally validate the model on a different but related independent data. In this study, we propose a permutation method to assess heterogeneity in external validation for risk prediction models. The permutation p value measures the extent of homology between development and validation datasets. If *p* < 0.05, the model may not be directly transported to the external validation population without further revision or updating. Monte-Carlo simulations are conducted to evaluate the statistical properties of the proposed method, and two microarray breast cancer datasets are analyzed for demonstration. The permutation method is easy to implement and is recommended for routine use in external validation for risk prediction models.

## Introduction

A risk prediction model estimates the probability that a certain outcome is present (diagnosis) or will occur (prognosis) in a new subject [1–3]. Once a prediction model has been constructed in a development population, the next step is to evaluate its prediction performance. This can be done by internal validation (e.g. bootstrapping [4] or cross-validation [5]), that is, constructing the model in one part (training dataset) and then evaluating its performance on another non-overlapping part (testing dataset) of the model development dataset.

Although internal validation can assess the *reproducibility* of a model, the value of a developed (diagnostic and prognostic) prediction model depends on its performance outside the development sample (*transportability*). The key is therefore to externally validate the model on a different but related independent data. Debray et al. [6] recently proposed a three-step framework to enhance the interpretation of external validation studies of prediction models. This should help researchers judge whether a prediction model is clinically practicable or merely statistically reproducible.

Following Debray et al.’s framework [6], we propose a permutation method to assess heterogeneity in external validation for risk prediction models. Monte Carlo simulation is implemented for the evaluation of our method. We demonstrate the application of the proposed method using two microarray breast cancer datasets.

## Methods

Suppose that a model development dataset (Data **D**) which consists of cases (subjects with the outcome) and controls (subjects without the outcome) is used to develop a prediction model (Model **M**). For external validation, Model **M** is tested on another independent validation dataset (Data **V**) to obtain a performance estimate: the externally validated AUC (area under the receiver operating characteristic curve), denoted as AUC^ext^.

To assess heterogeneity between Data **D** and **V**, we permute the subjects between these two datasets, separately for cases and controls. At the *j*th permutation, let **D** *_j_* and **V** *_j_* denote the permuted development and validation datasets, respectively. Data **D** *_j_* is used to develop a prediction model: **M** *_j_*. Data **V** *_j_* is then used to evaluate the performance of this Model **M** *_j_* to give a validated AUC, denoted as AUC*_j_*. The permutation process is repeated for a total of *k* times. The permutation *p* value is calculated as the proportion of the {AUC_1_, AUC_2_,..., AUC_*k*_} that are smaller than the previously calculated AUC^ext^.

The permutation *p* value measures the extent of homology between Data **D** and **V**. If the permutation *p* value is less than 0.05, we conclude that there is significant heterogeneity (at a significance level of *α* = 5%) between the two datasets. If the value is larger, we may transport the prediction model developed in Data **D** to Data **V**.

## Simulation Studies

### Simulation Setup

Suppose that there are three model development datasets (Data **D**_A_, Data **D**_B_, and Data **D**_C_), each with a different data structure. The variables in Data **D**_A_ and **D**_B_ are generated using the multivariate normal distributions for cases and controls, respectively (the means: detailed in S1 Exhibit; the variances: 1 for all variables; the correlation coefficients: 0 between any two variables in **D**_A_ and 0.2 between any two variables in **D**_B_). The variables in Data **D**_C_ are generated using a two component mixture of multivariate normal distributions for both cases and controls. (The variances for all variables are set to 1, and the correlation coefficients between any two variables, 0, for each component. Each component contributes 50% of the whole data. The means of these two multivariate normal distributions are detailed in S1 Exhibit.)

In each dataset, we use support vector machines (SVM) to construct a prediction model. SVM is an efficient learning algorithm for high-dimensional data in classification, regression and pattern recognition. The basis of SVM is to implicitly map data to a higher dimensional space via a kernel function to identify an optimal hyperplane that maximizes the margin between the two groups [7]. In this study, we use the e1071-packageof R with a default radial basis function kernel to obtain the prediction scores [8].

We consider three validation datasets (Data **V**_A_, Data **V**_B_, and Data **V**_C_). The data generating process and parameter setting for **V**_A_, **V**_B_, and **V**_C_ are the same as the aforementioned **D**_A_, **D**_B_, and **D**_C_, respectively. For homogeneity scenarios, we let the prediction models developed in **D**_A_, **D**_B_, and **D**_C_ be tested on **V**_A_, **V**_B_, and **V**_C_, respectively. For heterogeneity scenarios, we let the prediction model developed in one type of data be tested on a different type of data.

In the simulation, we consider prediction models with 3 and 10 predictors, respectively. We also consider three different sample sizes (small, medium, and large):*N* = 30 (cases)+30 (controls), 50+50, 100+100, respectively, for the model development datasets. We assume equal sample sizes for the development and the validation datasets. The number of permutations is set at *k* = 500, and the significance level is set at *α* = 0.05. We conduct a total of 5000 simulations for each scenario. In the simulation, we additionally create a very large validation dataset (1000 cases and 1000 controls) for each data type. These are used to determine a true AUC value for a prediction model as applied to the same model development population. We refer to these as the reproducibility AUCs.

### Simulation Results

Table 1 presents the results of homogeneity scenarios. We see that the externally validated AUCs and the corresponding reproducibility AUCs are approximately equal. We also see that the proposed permutation test has permutation *p* values that are around 0.5 and type I errors rates close to the nominal *α* level of 0.05.

**Table 1:** Results of the permutation analysis for the homogeneity scenarios.

**Sample size**	**3 predictors**				**10 predictors**			
	**Reproducibility AUC^a^**	**Externally validated AUC^b^**	**Permutation *p* value^c^**	**Type I error rate^d^**	**Reproducibility AUC^a^**	**Externally validated AUC^b^**	**Permutation *p* value^c^**	**Type I error rate^d^**
Development data: **T**_A_; Validation data: **V**_A_								
Small	0.7714	0.7739	0.4915	0.0530	0.7445	0.7425	0.5198	0.0496
Medium	0.7785	0.7854	0.5240	0.0460	0.7591	0.7590	0.5007	0.0476
Large	0.8065	0.7962	0.5001	0.0500	0.7724	0.7735	0.4875	0.0494
Development data: **T**_B_; Validation data: **V**_B_								
Small	0.7483	0.7627	0.4929	0.0598	0.6899	0.6916	0.5025	0.0542
Medium	0.7815	0.7761	0.4982	0.0496	0.7151	0.7098	0.5182	0.0488
Large	0.7871	0.7866	0.4953	0.0506	0.7247	0.7257	0.4988	0.0484
Development data: **T**_C_; Validation data: **V**_C_								
Small	0.7606	0.7715	0.4959	0.0464	0.7424	0.7515	0.5071	0.0454
Medium	0.7837	0.7836	0.5009	0.0562	0.7755	0.7650	0.5148	0.0520
Large	0.7951	0.7945	0.4934	0.0552	0.7731	0.7771	0.5221	0.0460

^a^: Performance for a prediction model as applied to the dataset which has exactly the same data structure as the model development population.

^b^: Performance of the model constructed in the development dataset and externally validate on another independent validation dataset.

^c^: Average permutation *p* values of 1,000 simulations.

^d^: Under the nominal α level of 0.05.

Table 2 presents the results of heterogeneity scenarios. We see that now the externally validated AUCs are smaller than the corresponding reproducibility AUCs. We also see that the permutation *p* value decreases when sample size increases and that the power (for detecting heterogeneity) of the permutation test increases when sample size increases.

**Table 2:** Results of the permutation analysis for the heterogeneity scenarios.

**Sample size**	**3 predictors**				**10 predictors**			
	**Reproducibility AUC^a^**	**Externally validated AUC^b^**	**Permutation *p* value^c^**	**Power^d^**	**Reproducibility AUC^a^**	**Externally validated AUC^b^**	**Permutation *p* value^c^**	**Power^d^**
Development data: **T**_A_; Validation data: **T**_B_								
Small	0.7679	0.6579	0.1685	0.4506	0.7337	0.6094	0.1958	0.3382
Medium	0.7790	0.6650	0.0887	0.6494	0.7471	0.6145	0.1016	0.5748
Large	0.7967	0.6723	0.0227	0.9002	0.7655	0.6174	0.0195	0.9076
Development data: **T**_A_; Validation data: **T**_C_								
Small	0.7765	0.6764	0.1936	0.4208	0.7235	0.6522	0.2090	0.3586
Medium	0.7804	0.6857	0.1198	0.6002	0.7573	0.6622	0.1394	0.5528
Large	0.7967	0.6919	0.0376	0.8458	0.7655	0.6695	0.0408	0.8342
Development data: **T**_B_; Validation data: **T**_A_								
Small	0.7748	0.6431	0.1595	0.5324	0.7005	0.6067	0.2085	0.3768
Medium	0.7834	0.6499	0.0743	0.7258	0.7202	0.6075	0.1162	0.6128
Large	0.7920	0.6508	0.0212	0.9306	0.7102	0.6098	0.0245	0.8868
Development data: **T**_B_; Validation data: **T**_C_								
Small	0.7732	0.6565	0.1710	0.4932	0.6901	0.6210	0.2377	0.3448
Medium	0.7802	0.6619	0.0935	0.6812	0.7230	0.6249	0.1473	0.5472
Large	0.7920	0.6714	0.0273	0.8910	0.7102	0.6298	0.0413	0.8320
Development data: **T**_C_; Validation data: **T**_A_								
Small	0.7680	0.6753	0.1883	0.4244	0.7652	0.6485	0.1998	0.3898
Medium	0.8000	0.6834	0.1135	0.5990	0.7652	0.6589	0.1149	0.5928
Large	0.8137	0.6912	0.0315	0.8456	0.7789	0.6672	0.0349	0.8566
Development data: **T**_C_; Validation data: **T**_B_								
Small	0.7671	0.6664	0.1830	0.4434	0.7522	0.6186	0.1980	0.3428
Medium	0.7762	0.6739	0.1170	0.6276	0.7756	0.6239	0.1051	0.5768
Large	0.8137	0.6840	0.0324	0.8640	0.7789	0.6283	0.0199	0.9052

^a^: Performance for a prediction model as applied to the dataset which has exactly the same data structure as the model development population.

^b^: Performance of the model constructed in the development dataset and externally validate on another independent validation dataset.

^c^: Average permutation *p* values of 1,000 simulations.

^d^: Under the nominal *α* level of 0.05.

## Real Data Application

Two independent microarray breast cancer datasets, W (Wang *et al*. [9]) and S (Sotiriou *et al*. [10]),were used to demonstrate the proposed method. The gene expression data and patient profile are available at the Gene Expression Omnibus database (http://www.ncbi.nlm.nih.gov/geo) with accession code GSE2034 (Data W) and GSE2990 (Data S). Both datasets were generated from the same Affymetrix-HG-U133A microarray platform. In the study of Wang *et al.* [9], Data W (consisting of 107 breast cancer patients with distant relapse and 197 without distant relapse) was divided into training (115 patients) and testing (171 patients) by concentration of the estrogen receptor, and a 76-gene signature was identified with an *internally* validated AUC of 0.694. Here we use Data S (consisting of 120 breast cancer patients without relapse and 67 with relapse; 2 patients with unknown relapse status are omitted from our analysis) to validate the prediction performance of the 76-gene signature developed in Data W, and the *externally* validated AUC is 0.534.

Next, we conduct the permutation test. We performed a total of 100,000 permutations and found that all the permuted AUCs are larger than 0.534 (permutation *p* value < 10^−5^). Hence we conclude a significant heterogeneity between Data W and S. For this example, we know that the 76-gene signature developed in Data W cannot be directly transported to Data S, unless further model updating or revision was done.

## Discussion

Debray et al. [6] suggested using the following three steps to interpret the results of external validation of a prediction model: 1) to assess the extent of relatedness between development and validation datasets, 2) to assess the performance of the model on the external validation dataset, and 3) to interpret the model’s predictive accuracy given the results from 1) and 2). Our permutation method integrates the above steps 1 and 2. The permutation *p* value measures the extent of homology between development and validation datasets (step 1), while at the same time the homology/heterogeneity judgment is based directly on model performance comparison between development and validation datasets (step 2). This should greatly facilitate the interpretation of external validation studies of prediction models.

If the purpose of the model is purely to make predictions for new individuals in the same population or future patients in the same clinical setting (the temporal validation [11]), then we need a model that has good reproducibility. To estimate the reproducibility AUC, one can use an internal validation method, or better still, to sample more subjects in the same population for an ‘external’ validation; external here to be taken relative to the model development data at hand but not to the study population at large. Our permutation method can be applied in this situation to help check whether there is significant temporal variation in case-mix in the population that will curtail the utility of the prediction model.

But more often, the purpose of the model is for making predictions for subjects *outside* the model development population. We encourage the model developers to pursue as many external datasets as possible to validate the model, if transportability of the model is intended. Here the permutation *p* value from our proposed permutation test is a measure of homology between a chosen external dataset and the model development dataset. If the permutation *p* value of an external dataset from a certain population is less than 0.05, there is significant heterogeneity between the two datasets and the model may not be directly transported to that external population without further revision or updating [12,13].

In summary, the value of a developed prediction model depends on its performance outside the development sample. The permutation method proposed in this paper assesses heterogeneity in external validation for risk prediction models by integrating the step 1 and step 2 of Debray et al.’s three-step framework [6]. This should greatly facilitate the interpretation of external validation studies of prediction models. The method is easy to implement and is recommended for routine use in external validation for risk prediction models.

## Supporting Information

S1 Exhibit The means of the three model development datasets Data **D**_A_, Data **D**_B_, and Data **D**_C_ in simulation studies. (DOCX)
