# Supplementary material for: A Permutation Method to Assess Heterogeneity in External Validation for Risk Prediction Models
Source: PLoS One. 2015 Jan 21;10(1):e0116957. doi: 10.1371/journal.pone.0116957 (PMC4301917; doi:10.1371/journal.pone.0116957)
Supplement: S1 Exhibit — (DOCX) [file pone.0116957.s001.docx]

Exhibit S1. The means of the three model development datasets in simulation studies: Data $D_{\text{A}}$, Data $D_{\text{B}}$, and Data $D_{\text{C}}$.

For Models with 3 Predictors

| Dataset | Variable 1 | Variable 2 | Variable 3 |
| --- | --- | --- | --- |
| Data $D_{\text{A}}$ | 1.0 | 0.5 | 0.7 |
| Data $D_{\text{B}}$ | 0.1 | 1.2 | 0.6 |
| Data $D_{\text{C}}$ |  |  |  |
| Component 1 | 0.7 | 0.8 | -0.4 |
| Component 2 | 0.9 | 1.2 | -0.3 |

For Models with 10 Predictors

| Dataset | Variable 1 | Variable 2 | Variable 3 | Variable 4 | Variable 5 | Variable 6 | Variable 7 | Variable 8 | Variable 9 | Variable 10 |
| --- | --- | --- | --- | --- | --- | --- | --- | --- | --- | --- |
| Data $D_{\text{A}}$ | 0.15 | 0.25 | 0.30 | 0.35 | 0.40 | 0.45 | 0.50 | 0.60 | -0.10 | 0.65 |
| Data $D_{\text{B}}$ | 0.10 | 0.30 | 0.35 | 0.40 | 0.45 | 0.50 | 0.60 | -0.30 | -0.20 | 0.15 |
| Data $D_{\text{C}}$ |  |  |  |  |  |  |  |  |  |  |
| Component 1 | 0.65 | 0.70 | 0.80 | 0.90 | 0.40 | 0.35 | 0.55 | 0.25 | 0.40 | -0.50 |
| Component 2 | -0.20 | 0.10 | 0.25 | 0.30 | 0.35 | 0.6 | 0.45 | 0.40 | 0.25 | -0.35 |
